# Application and Revision of Montreal Cognitive Assessment in China's Military Retirees with Mild Cognitive Impairment

**DOI:** 10.1371/journal.pone.0145547

**Published:** 2016-01-04

**Authors:** Yali Zhai, Qiuling Chao, Hong Li, Bo Wang, Rong Xu, Ning Wang, Yajun Han, Xiaole He, Xin Jia, Xiaoming Wang

**Affiliations:** 1 Department of Geriatrics, Xijing Hospital, Fourth Military Medical University. Xi’an, Shaanxi, China; 2 Center for Ageing and Health Research, Xi’an Jiaotong University, Xi’an, Shaanxi, China; 3 Department of Epidemic, Fourth Military Medical University, Xi’an, Shaanxi, China; Nanjing University of Aeronautic and Astronautics, CHINA

## Abstract

**Objective:**

In an effort to accommodate MOCA to better fit for the Chinese context, this study was designed to employ the MOCA criteria to screen mild cognitive impairment (MCI) and analyze associated risk factors in military retirees.

**Methods:**

Three hundred and four retired military cadres were recruited using a random cluster sampling technique with information collected including personal, prevalence, MOCA scale, and related neuropsychiatry scale. Thirty retirees were randomly chosen to be further analyzed one month later using the revised MOCA scale.

**Results:**

①Our data indicated an incidence rate of 64.8% for mild cognitive impairment in retired military cadres. The incidence rate for MCI was significantly higher in those aged 80 or above compared with those 80 years of age or younger (P<0.05). The incidence rate of MCI was significantly higher in those with fewer than 6 years of education compared with those with over 7 years of education (P<0.05). The MCI incidence was higher for those with little exercise than those taking regular exercise (P<0.01). Moreover, the MCI incidence was higher in stroke patients than those who never had a stroke episode (P<0.05). ②There was a significant correlation between MOCA and MMSE scale scores (r = 0.81). MOCA scale scores were negatively correlated with ADL and CES-D scores (although not PSQI scores). ③ MOCA recension Cronbach’s alpha value was 0.862. The related coefficient of MOCA and MOCA recension was 0.878(P<0.01). When the Score of cut-off -point of the MOCA recension was 28, the area in ROC curve analyses was 0.859, as well as the largest area.

**Conclusion:**

Retired cadres exhibited a greater incidence of MCI (than general population), which was closely associated with age, level of education and physical exercise and cerebral apoplexy. Revised MOCA scale displays a better validity and reaction degree of reliability and is more suitable for screening and diagnosis of MCI in the elderly in China.

## Background

The prevalence of dementia is estimated to be as high as 24 million globally. It is estimated that cases of dementia will double every 20 years through 2040, resulting in a huge health care burden. Alzheimer disease (AD), a leading cause of dementia, is characterized by a progressive decline in cognitive function, which typically begins with deterioration in memory[1]. AD represents the sixth cause of mortality of all ages in the United States, and ranks the fifth for the cause of death for those 65 years of age or older (Alzheimer’s Association 2011) [3]. AD is associated with a health-care cost of US$172 billion per year[2]. AD is usually divided into three stages, presymptomatic, early symptomatic (or mild cognitive impairment) and dementia. In presymptomatic stage, specific biomarkers are often available while it is typically ignored due to absence of symptoms. Mild cognitive impairment stage is characterized by memory and functional loss which is the optimal stage for preventive and interventional therapies. It is thus crucial to detect mild cognitive impairment early for the screening and intervention of AD occurrence.

Mild cognitive impairment (MCI) is widely regarded as the intermediate stage of cognitive impairment between changes in normal cognitive aging and those associated with dementia. Elderly MCI patients constitute a high-risk population for the onset and development of dementia, in particular AD[4]. Initially being referred to as a specific stage of cognitive deterioration identified through the Global Deterioration Scale(GDS) in 1980s[5]. the operational definition of MCI has undergone several updates over the last decade and remains as an evolving issue for diagnosis[6–9].

In subsequent years, the criteria for MCI were revised to encompass other patterns of cognitive impairment in addition to memory loss. The MCI Working Group of the European Consortium on Alzheimer’s Disease renewed the criteria for MCI in 2005[10], In 2011 the National Institute on Aging-Alzheimer’s Association (NIA-AA) workgroup proposed criteria specifically for MCI due to AD for both clinical and research settings [11] as follows: 1). Concerning regarding a change in cognition self/informant/clinician report;2). Objective evidence of impairment in one or more cognitive domains, typically including memory; 3). Preservation of independence in functional abilities; 4).Not demented. According to the type and number of affected cognitive domains, the MCI can be classified into different types[12].

The global prevalence of MCI in the elderly is estimated to be 15–20% [13]. The diagnosis of MCI is associated with a higher rate of progression to AD on follow-up, and the rate of conversion to AD was around 48.7% in subjects presenting with amnestic MCI[14]. There are also a large number of subjects classified as MCI which can resume normal cognitive function or maintain stable cognitive deficits, without progressing into dementia even with long-term follow-ups [15].

The clinical procedures for the diagnosis of MCI are rather complex and, in many instances, cognitive deficits are very mild, Under such scenario, a comprehensive neuropsychological evaluation may be considered a gold standard for the identification of patients with MCI[10]. However, formal neuropsychological testing is time-consuming, expensive and not readily available. Furthermore, such test requires highly trained and skilled professionals to perform, thus making it pertinent to develop strategies that are cost-effective and easy to administer with results easy to be interpreted.

The MOCA (Montreal Cognitive Assessment) is a brief cognitive test specifically developed to screen for mild cognitive deficits and has been regarded as a suitable test for initial workup of subjects with suspected MCI[16,17]. It was firstly developed in 1996 based on the clinical intuition of one of the authors (ZN) regarding domains of impairment commonly encountered in MCI and best adapted to a screening test[16]. Following the iterative modification, the final version of the MOCA is a one-page 30-point test administered in 10 minutes. The MOCA was administered in French and English as appropriate in 2005.

The mini-mental state examination (MMSE) was developed in 1975 as a brief test for the quantitative assessment of cognitive impairment in adults [18]. It is the most widely employed cognitive screening test in both clinical and research settings. Despite good sensitivity and specificity for diagnosis of dementia, its commonly used cutoff scores do not display good accuracy for differential diagnosis for MCI, with mistakenly identifying most subjects with normal cognitive function.

The specificity of the MOCA to exclude elderly normal controls was good (87%), although slightly lower than the MMSE. More important, the MOCA’s sensitivity in detecting MCI was excellent (90%), and it was considerably more sensitive than was the MMSE. The MOCA also detected mild AD with high sensitivity (100%) and excellent specificity(87%)[12].

Given its high sensitivity and specificity in quickly screening/detecting MCI[16,19–22]. The MOCA scale has been widely employed worldwide. While due to the heavy cultural dependence it has been extensively revised in various countries. For the apparent difference in culture and lifestyle between China and the west, considering atypical illustrations, obscure words, and lacking of apprehension, MOCA requires further evaluation and perhaps revision to adapt to Chinese better. A better illustration of the MOCA protocol should help to minimize unnecessary controversy and difficulty in the Chinese elderly [23–25].

This study was designed to employ MOCA to examine mild cognitive impairment and to analyze related epidemiological risk factors in Xi’an retired military cadres. what’s more, we hope to further modify MOCA in clinical practice to fit the Chinese cultural background, living habits and experience as well as improve its reliability and validity for Chinese population.

## Object and Methods

### Object

Between January and August in 2011, a survey was conducted among military retirees from 9 Xi’an military sanatoria. A randomized cluster sampling was employed. A signed written consent form was obtained from all participants. Three hundred and twenty-six questionnaires were sent out and three hundred and twelve of which were returned, including three hundred and four completed the study, reflecting a recovery rate and effective rate of 95.7% and 97.4%, respectively. One hundred and ninety-six military retired cadres were surveyed with the revised MOCA scale at the same time. These cadres include one hundred and eighty- one men and fifteen women with an average age of 81±4 who received an average of 9.6±4.6 years of education. Thirty military retired cadres were randomly chosen for a follow-up test one month later.

### Investigation protocols

1. **State of health investigate scale.** The scale categories include name, age, sex, number of years of education, level of education, exercise, hobby, history of chronic disease (including coronary heart disease, hypertension, stroke, diabetes, chronic obstructive pulmonary disease). All chronic diseases were diagnosed using respective diagnostic criteria.
2. **Montreal cognitive assessment (MOCA) scale.** The scale’s total score is 30. A score of 26 or less denotes presence of mild cognitive impairment (MCI). MOCA scale is a one-page 30-point test administered in 10 minutes. Details on the specific MoCA are as follows. The short-term memory recall task(5 points) involves two learning trials of five nouns and delayed recall after approximately 5 minutes. Visuospatial abilities are assessed using a clock-drawing task(3 points) and a three-dimensional cube copy(1 point). Multiple aspects of executive functions are assessed using an alternation task adapted from the Trail Making B task(1 point), a phonemic fluency task(1 point), and a two-item verbal abstraction task(2 points). Attention, concentration, and working memory are evaluated using a sustained attention task(target detection using tapping;1 point), a serial subtraction task(3 points), and digits forward and backward(1 point each). Language is assessed using a three-item confrontation naming task with low-familiarity animals (lion, camel,rhinoceros;3 points), repetition of two syntactically complex sentences(2 points), and the aforementioned fluency task. Finally, orientation to time and place is evaluated(6 points).
3. **Mini-mental state examination (MMSE) scale.** The scale’s total score is 30. Based on the level of education, it is identified a score of 20 or below as illiterate (less than 1 year of education), a score between 23 and 27 as primary (education years from 1 to 6 years), and a score of 27 or higher as middle or above (education years more than 7 years) [26].
4. **Activities of daily living (ADL) scale.** It is identified that a score of 26 or higher as abnormal.
5. **Center for epidemiological survey, depression Scale(CES-D) scale.** It is identified as ‘not depressed’ with a score of 15 or lower, ‘probably depressed’ with a score between 16 and 19, and definitely depressed with a score of 20 or higher.
6. **Pittsburgh sleep quality index (PSQI) scale.** The scale’s total score ranges from 0 to 21. The higher the score, the worse the sleep quality. Sleep disorder is identified with a score of 7 or higher.

### The revised MOCA

#### Revised method

On the basis of preserving the main content of the original MOCA scale, we have access to a wide range of literature, solicit opinions from the neurologist deeply and add some content about language understanding in MMSE scale, then test on Chinese elderly patients in department of geriatrics repeatedly (Table 1).

**Table 1:** Comparison revised MOCA with original MOCA.

Revised details	original MOCA	revised MOCA
visual space	Alternate ligature test 1, 2, 3, 4, 5, A, B, C, D, E, according to increasing sequence from number to letter with line connect, connect and no accross.	Alternate ligature test From 1 to 10, odd number and even number with line connect according to increasing sequence, connect and no accross.
	Copy cube full line cube	Copy cube dotted line in cube
name	hand drawing: lion, camel, rhinoceros	photograph: dog, mouse, cock
short-term memory recall task	face, silk, church, daisy, red	blue sky, jacket, teacup, car, hospital
language	Repetition He have not come home from outside; He find the room full of people when he come back.	Repetition A group of children are playing in the garden surrounded by flowers and trees.
		Add reading, language understanding and writing Please pick up the book with your left hand and turn to page 69.Please write a complete meaningful sentence.
abstract	Words similarity train—bicycle; watch—ruler: What is the connection?	Words similarity banana, watermelon, apple, pumpkin; ship, car, factory, plane What is the odd one out?
time space orientation	date, month, year, week, place, city	year, month, date, city, place

#### Revised details

The MOCA is revised to fit the Chinese cultural backgrounds, living habits and experiences, supplements the reading, language understanding and writing portion which differentiates with the original MOCA.

### Investigation method

Three geriatric physicians and six primary nurses were chosen as investigators following a short neuropsychological screening scale training and examination. The investigation was performed with individual interviews. Following collection of the questionnaires, three investigators summarized and scored the investigate scales together to ensure integrity and accuracy of the contents and the scores.

### Quality control measures

In order to improve the patients’ cooperation, a brief physical examination and health consultation were offered to the participants. Upon completion of the survey, questionnaire was examined for integrity and accuracy daily. Incorrect or uncompleted information was revised in a timely fashion. At the stage of data entry, two investigators were chosen to enter all data independently to ensure integrity and accuracy.

### Ethics

The study was operated in accordance with the World Medical Association’s Declaration of Helsinki. The study was approved by the ethics committee of the Fourth Military Medical University. All eligible patients are informed verbally and in writing about the aim and practical carrying out of the trial besides their rights as participants. All participants signed written informed consent forms prior to randomization. All data was handled with confidentiality, and the patients were ensured anonymous. The trial is registered on CilinicalTrials.gov (NCT 02483754).

### Data processing and analysis

EpiData3.0 software was applied to establish database, The data was analyzed using SPSS13.0 software. The revised MOCA was analyzed using the following tests: viability test, correlation analysis and degree of reaction analysis with means of Chi-square test, Pearson relative, variance analysis. Differences between values were considered statistically significant when p< 0.05.

## Result

### MOCA check MCI occurrence rate

Among the 304 participants recruited, 197 of which had a MOCA total score below 26, 64.8% incidence of mild cognitive impairment in Xi’an military retirees.

### Analysis of related risk factors with MOCA screening MCI

As showed in Table 2, the incidence rate of MCI was higher in the group aged 80 or higher (67.56%) compared with that of 70 years of age (47.62%) (P<0.05). The incidence rate of MCI was 72% for those less than 6 years of education compared with those over 7 years of education (P<0.05). The MCI incidence rate was 80.95% for those who had little exercise compared with those taking regular exercise (60.58%) (P<0.01). The MCI incidence was 73.33% in stroke patients compared with those without stroke history (57.64%) (P<0.05). The incidence rate of MCI displayed slightly impact for gender, personal hobbies, coronary heart disease, hypertension, diabetes, chronic obstructive pulmonary disease, peptic ulcer, retrogressive osteoarticular diseases.

**Table 2:** Analysis of related risky factors with MCI.

variable	MCI		normal		x^2^	P
	n	%	n	%		
age						
70~79	20	47.62	22	52.38		
80~	177	67.56	85	32.44	6.31	0.01
sex						
male	188	65.51	99	34.49		
female	9	52.94	8	47.06	1.11	0.29
education years						
<6	78	75.43	25	24.27		
7~12	75	60.48	49	39.52		
>12	44	57.14	33	42.86	8.38	0.015
exercise						
yes	146	60.58	95	39.42		
no	51	80.95	12	19.05	9.08	0.00
hobbies						
yes	190	64.19	106	35.81		
no	7	87.50	1	12.50	1.86	0.17
chronic disease						
CHD						
yes	138	66.67	69	33.33		
no	59	60.82	38	39.18	0.99	0.32
hypertension						
yes	121	66.12	62	33.88		
no	76	62.81	45	37.19	0.35	0.55
stroke						
yes	58	74.36	20	25.64		
no	139	61.50	87	38.50	4.20	0.04
diabetes						
yes	58	70.73	24	29.27		
no	139	62.61	83	37.39	1.73	0.19
COPD						
yes	25	71.43	10	28.57		
no	172	63.94	97	36.06	0.76	0.38
peptic ulcer						
yes	13	54.17	11	45.83		
no	184	65.71	96	34.29	1.29	0.26
ROAD						
yes	26	74.29	9	25.71		
no	171	63.57	98	36.43	1.56	0.21

CHD, coronary heart disease; COPD, chronic obstructive pulmonary disease; ROAD, retrogressive osteoarticular disease

### Correlation analysis of MOCA scale score with MMSE, ADL, CES-D and PQSI

It shows that MOCA score is significantly correlated with MMSE score (with a r value of 0.81) in Table 3. MOCA score displayed a negative correlation with ADL and CES-D. Our data revealed that the higher the MOCA score and the better the ADL, the lower the possibility of occurrence of depression. MOCA score exhibited no correlation with PQSI.

**Table 3:** Correlation analysis of MOCA scale score with MMSE, ADL, CES-D and PQSI.

		MMSE score	ADL score	CES-D score	PQST score
MOCA	relativity ratio score	0.81	-0.49	-0.24	0.05
	P	0.00	0.00	0.00	0.42

### Revised MOCA analysis of reliability and validity

#### Reliability

MOCA recension Cronbach’s alpha is 0.862.Two-part reliability coefficient is 0.862. Correlation between forms scale scores in the correlation coefficient is 0.669. Thirty military retired cadres were selected randomly and were tested again after one month, and the test of 0.831 demonstrates a better reliability.

#### Validity

*Criterion validity analysis*: Our results in Table 4 revealed that the revised MOCA and the original test displayed significant correlation (0.308 ~ 0.942, P<0.05). The related coefficient of MOCA and MOCA recension is 0.878(P<0.01).

**Table 4:** Criterion validity analysis of revised MOCA and original MOCA.

	visual space	name	language	delayed memory	attention	abstract	time space orientation
visual space	0.942**						
name		0.412**					
language			0.512**				
delayed memory				0.580**			
attention					0.864**		
abstract						0.308**	
time space orientation							0.950**

The horizontally items represent the original data, the vertically items represent the revised data.

**P<0.01

*Internal validity*: The revised MOCA factors and total score exhibited significant correlation (Table 5), with a correlation coefficient between 0.392 and 0.832 (P<0.01).

**Table 5:** Correlation analysis of factor points and total score in revised MOCA.

Item	Correlation coefficient	P
visual space	0.832	0.000
Name	0.392	0.000
Language	0.773	0.000
delayed memory	0.715	0.000
Attention	0.820	0.000
Abstract	0.646	0.000
time space orientation	0.600	0.000

*Degree of reaction analysis*: The total score of the test displayed significant variation in visual space, language, delayed memory, attention and abstraction in different age groups (P<0.05) (Table 6). Aging imposes as a negative factor for the score. Men and women displayed a significant difference in visuospatial executive function and delayed memory ability (P<0.05). Females exhibited better visuospatial executive function and delayed memory ability compared with male counterpart. The results also varied in executive function of visual space, language, attention, space capacity of orientation and education (P<0.05). Less than six years of education and six to twelve years of education is different in visuospatial executive function, language, attention and orientation. Less than six years of education displayed significant difference in visuospatial executive function, language, attention, abstract and orientation compared with those over 12 years of education.

**Table 6:** Correlation analysis of total score and factor points in MOCA with age, gender and education.

category		n	visual space	name	language	delayed memory	attention	abstract	diecrtation	score
agesexed	＜	104	3.78±1.3	2.91±0.42	3.80±0.6^a^3.	3.86±1.2^a^3	5.46±1.1^a^	1.95±0.3^a^	4.88±0.54.66	26.6±3.9^a^2
ucated	80≥80m	921	^a^3.37±1.73	.88±0.52.	42±0.93.6	.15±1.6	5.01±1.4	1.69±0.	±1.14.76±0.	4.2±6.325
time	anwome	811	.52±1.5	89±0.53.0	0±0.83.87	3.5±1.5	5.21±1.3	71.82±0	95.0±0.04.4	.3±5.5^b^25
	n<66~1	559	^b^4.47±0.82	±0.02.86±	±0.53.31±	^b^4.3±1.0	5.73±0.7	.52.0±0.	7±1.24.86±	.5±5.323.
	2>12	884	.80±1.53.8	0.52.90±0	1.33.69±0.	3.20±1.	4.7±1.75	01.69±0	0.8^c^4.98±0.	0±6.426.0
		9	0±1.3^c^4.18	.52.94±0.	7^c^3.88±0.	63.56±1	.32±1.2^c^	.71.84±	1^d^	±4.9^c^27.6
			±1.3^d^	4	4^d^	.43.86±1.4	5.80±0.5 ^d^	0.51.98		±3.0^d^

^a^*P*<0.05, ＜80 years *vs* ≥80 years;

^b^*P*<0.05, men *vs* women;

^c^*P*<0.05, <6 years *vs* 6~12 years in educated time;

^d^*P*<0.05, <6 years *vs* >12 years in educated time.

### Score of cut-off-point

The ROC curve is drawn with revised MOCA in the MOCA scale (Chinese version) test results as the standard (S1 Fig). When the Score of cut-off -point of the MOCA recension was 28, the area in ROC curve analyses was 0.859(0.809~0.910), as well as the largest area.

### Diagnostic accordance rate

We repeated the survey using revised MOCA and original MOCA. Both MOCA scale diagnosed 63 patients with mild cognitive impairment, and 78 objects as normal. The diagnostic accordance rate was 72.68%.

## Discussion

Alzheimer’s disease is an age-related neurodegenerative process. According to Ferry [27], it is reported that about 80% people aged 65 or above are prone to the development of Alzheimer’s disease. Studies have shown that elderly patients with MCI are at a high risk of dementia. These individuals develop AD faster than the general elderly population. On average, 10%~15% MCI patients envenually develop Alzheimer’s disease (AD) each year [28]. However, prevalence of MCI may vary around the world. For example, the MCI prevalence rate is estimated to be 4.9%~22.2% in the elderly (60 years or above) [29–32]. Ana Luisa Sosa researched that the prevalence of aMCI ranged from 0.8% in China to 4.3% in India [33]. Chinese scholars revealed that the MCI prevalence rate in the elderly is 4.5%~71.74% in China [34–38]. This study reveales that the MCI prevalence rate in military retirees from China was higher than that in the world. One of the main reasons may be related to older age (81±4 years) and poor education (average education year: 9.6±4.6), and difficulty in understanding the contents by Chinese elderly in MOCA.

Although MCI may be dependent upon many factors, ample of evidence has shown that age may be the primary risk factor for MCI. ZHANG and colleagues [38] showed that 74.17% individuals with cognitive dysfunction in Chinese urban areas are between the ages of 85 and 90. Ramlall’s [39] study showed that MCI was associated with increasing age and low education levels. Petersen’s [30] results of the binary logistical regression analysis indicated that age and history of stroke were associated with MCI in men. For women, the risk factors were lower level of education and lack of religious attendance. Severe white matter lesions (WML) significantly increases the risk of developing to MCI over a 7-year period in low educated participants [40]. Many articles and reviews show that cardiovascular risk factors (CVRFs) have been considered to serve as risk factors for cognitive decline and AD [41–43]. In many cases, risk markers for vascular cognitive impairment (VCI) are the same as traditional risk factors for stroke [44]. Recurrent stroke is one of higher risk factors for dementia [45]. 10% of patients developed new dementia soon after the first stroke, and more than a third had dementia after recurrent stroke [46]. In a community-based study of stroke done in Rochester, MN, the prevalence of dementia was 30% immediately after stroke, and the incidence of new-onset dementia increased from 7% after 1 year to 48% after 25 years [47]. However, clinical studies have shown that subjects with VaMCI can present with a broader cognitive impairment, which can also include memory deficits [48]. The neuropathology of cognitive impairment in later life is often a mixture of Alzheimer disease and microvascular brain damage, which may overlap and synergize the risk of cognitive impairment. Patients with poststroke dementia (PSD) have degrees of functional impairment and high mortality rates [49]. In stroke cases, subjective and objective cognitive performance predicts dementia. Identifying individuals with stroke at the greatest risk of dementia has important implications for treatment and intervention [50]. However, detection and control of the traditional risk factors for stroke and cardiovascular disease may be effective in the prevention of VCI, even in older people.

In recent studies, three lifestyle factors are believed to contribute in slowing down the rate of cognitive decline and dementia, namely socially integrated network, cognitive leisure activity, and regular physical activity. Among these three factors, physical activity is deemed the most important lifestyle factor to protect against deleterious sequelae of aging on health and cognition [51–53]. Other studies also suggest a role for physical activity in significantly and consistently improving functional impairment and cognition [54, 55]. Habitual physical activity (HPA) status is associated with executive performance in AD population and benefits AD patients [56]. Geda and colleagues [57] observed that moderate activity during midlife was associated with a 39% reduction in the risk of mild cognitive impairment later on in life. Moderate exercise during late-life was associated with a 32% lower risk for MCI. Late-life depression is a strong risk factor for progression from normal to MCI, and a borderline-significant risk factor for the progression from MCI to AD [58]. Depression increases risk for later mild cognitive impairment and predicts amnestic mild cognitive impairment [59]. Depression is a stronger risk factor than virtually all AD risk factors that have emerged from large epidemiological studies in well-characterized cohorts [60]. Depression is associated with changes in activity of frontal and limbic circuits [61]. Symptoms of depression may reflect pathophysiological changes due to AD pathology, as amyloid deposition in brain occurs a decade or longer before cognitive symptoms become apparent [62]. The results of this study are in the accordance with those mentioned above.

Both MMSE and MOCA are most widely used in screening and diagnosis of mild cognitive impairment (MCI). ADL and CES-D are often used in the diagnosis and evaluation of patients with MCI. The impaired instrumental activities of daily living (IADL) in MCI group display a more widespread pattern of gray matter loss involving frontal and parietal regions, worsened episodic memory and executive functions, and a higher percentage of individuals progressing to AD than the relatively intact IADL MCI group [63]. This research show that in screening of MCI patients, MOCA score has a significant correlation with MMSE score and a negative correlation with ADL and CES-D. That is to say, the higher MOCA score and the better the cognitive function and daily activity function, the lower the possibility of depression.

Because of the different cultural background and lifestyles, MOCA can’t fully fit and reflect the level of cognitive dysfunction in diagnosis and screening of the elderly in China. The MOCA which has been revised according to the culture and lifestyle of Chinese is more likely to be adopted by these subjects, with a more valid test result. Through statistical analysis, it has been shown that the revised MOCA scale has good reliability, validity and reaction degrees which are more suitable for screening and evaluation MCI in Chinese elderly people.

Our research aims at the shortcomings of the translated MOCA version existing in China and revises it firstly, although we take sample from a particular group, which can’t represent the Chinese population as a whole, we overcome the deviation due to the difference between Chinese and west cultural backgrounds, making a basement for broadening the research. At the same time, this study uncovers the MCI prevalence of retired military cadres in Chinese, indicates a great reliability and validity with the revised MOCA which can be used as a initial screening tool for MCI as well as popularized in different areas and larger population in China further.

## Supporting Information

S1 Fig The ROC curve of revised MOCA in elderly people with mild cognitive impairment. (TIF)
