# Correction: Application and Revision of Montreal Cognitive Assessment in China's Military Retirees with Mild Cognitive Impairment

**DOI:** 10.1371/journal.pone.0149825

**Published:** 2016-02-12

**Authors:** Yali Zhai, Qiuling Chao, Hong Li, Bo Wang, Rong Xu, Ning Wang, Yajun Han, Xiaole He, Xin Jia, Xiaoming Wang

There is an error in the Background section of the manuscript. The references are swapped. Reference [2] should be [3] and vice versa.

There is an error in the subsection Investigation Protocols of the section Object and Methods. The fourth sentence of the paragraph titled “Montreal cognitive assessment (MOCA) scale” is incorrect. The correct sentence is: Details on the specific MOCA are as follows.

There is an error in Table 6. The rows were formatted incorrectly. Please see the corrected version here.

**Table 6:** Correlation analysis of total score and factor points in MOCA with age, gender and education

category		n	visual space	name	language	delayed memory	attention	abstract	diecrtation	score
age	<80	104	3.78±1.3 ^a^	2.91±0.4	3.80±0.6^a^	3.86±1.2^a^	5.46±1.1^a^	1.95±0.3^a^	4.88±0.5	26.6±3.9^a^
	≥80	92	3.37±1.7	2.88±0.5	3.42±0.9	3.15±1.6	5.01±1.4	1.69±0.7	4.66±1.1	24.2±6.3
sex	man	181	3.52±1.5 ^b^	2.89±0.5	3.60±0.8	3.5±1.5 ^b^	5.21±1.3	1.82±0.5	4.76±0.9	25.3±5.5^b^
	women	15	4.47±0.8	3.0±0.0	3.87±0.5	4.3±1.0	5.73±0.7	2.0±0.0	5.0±0.0	25.5±5.3
educated time	<6	59	2.80±1.5	2.86±0.5	3.31±1.3	3.20±1.6	4.7±1.7	1.69±0.7	4.47±1.2	23.0±6.4
	6~12	88	3.80±1.3^c^	2.90±0.5	3.69±0.7^c^	3.56±1.4	5.32±1.2^c^	1.84±0.5	4.86±0.8^c^	26.0±4.9^c^
	>12	49	4.18±1.3^d^	2.94±0.4	3.88±0.4^d^	3.86±1.4	5.80±0.5 ^d^	1.98±0.4^d^	4.98±0.1^d^	27.6±3.0^d^

^a^*P*<0.05, <80 years *vs* ≥80 years

^b^*P*<0.05, men *vs* women

^c^*P*<0.05, <6 years *vs* 6~12 years in educated time

^d^*P*<0.05, <6 years *vs* >12 years in educated time.
